# The role of autoimmunity in premature ovarian failure

**Published:** 2015-08

**Authors:** Mahbod Ebrahimi, Firouzeh Akbari Asbagh

**Affiliations:** *Department of Obstetrics and Gynecology, Moheb-e-Yas Women General Hospital, Faculty of Medicine, Tehran University of Medical Sciences and Health Services, Tehran, Iran.*

**Keywords:** *Premature ovarian failure*, *Premature ovarian insufficiency*, *Autoimmune oophoritis*, *Autoimmunity*

## Abstract

Premature ovarian failure (POF) is a heterogeneous syndrome with several causative factors. Autoimmune mechanisms are involved in pathogenesis of 4-30 % of POF cases. The present review focuses on the role of autoimmunity in the pathophysiology of POF. The evidences for an autoimmune etiology are: demonstration of ovarian autoantibodies, the presence of lymphocytic oophoritis, and association with other autoimmune disorders. Several ovarian antigenic targets have been identified in POF patients. The oocyte seems to be the most often targeted cell. Lymphocytic oophoritis is widely present in POF associated adrenal insufficiency. Addisonۥs disease is one of the most common autoimmune disorders associated with POF. Early detection of this potentially life threatening disease was recommended in several studies.

The gold standard for detecting autoimmune POF is ovarian biopsy. This procedure is not recommended due to unknown clinical value, expense, and risks. Several immunoassays have been proposed as substitute diagnostic tools. Nevertheless, there is no clinically proven sensitive and specific serum test to confirm the diagnosis of autoimmune POF or to anticipate the patient’s chance of developing POF or associated diseases.

Some authors suggested the possible effects of immuno-modulating therapy on the resumption of ovarian function and fertility in a selected group of autoimmune POF patients. However, in most instances, this treatment fails to reverse the course of the disease.

Numerous studies illustrated that standard treatment outcome for infertility is less effective in the presence of ovarian autoimmunity. The antibody-induced damage could be a pathogenic factor. Nevertheless, the precise cause remains obscure.

## Introduction

Premature ovarian failure (POF) is a term to define the women younger than 40 years of age who present with amenorrhea lasting more than 4 months and hypoestrogenic-hypergonadotropic serum profile (follicle stimulating hormone (FSH) levels≥ 40 mIU/mL on two occasions) (1-5). This disorder is characterized by anovulation, amenorrhea, sex steroid deficiency, and infertility (1). The most common presentation is secondary amenorrhea and the main consequences are infertility and psychological stress (6-8). However, POF is an important cause of secondary amenorrhea and infertility, some patients may continue to ovulate and conceive (7-10). Therefore, "premature ovarian insufficiency" and "premature ovarian dysfunction" are more accurate phrases to reflect reversible nature of this condition (11-14). The disease affects 0.3-1% of general population (6, 15-17). Follicle depletion and follicle dysfunction are two main etiological mechanisms (9, 18). Follicle depletion can be the consequence of a primitive reduced pool of oocytes or an accelerated follicular atresia (10). FSH-receptor mutation, inappropriate luteinization related to low follicular count, and autoimmune oophoritis were suggested as the causes of follicle dysfunction (9, 19). The etiology of the disease varies in different patients: chromosomal/genetic abnormalities, metabolic/enzymatic factors, autoimmunity, infections, environmental toxins, and iatrogenic influences involve in development of the disease (1, 10, 20-22). Nevertheless, the precise cause is undetermined in a significant portion of patients. These cases were classified as idiopathic (2, 23-28). Based on clinical observations, immunological data, and histological findings, autoimmunity may be the pathogenic mechanism in 30% of cases of idiopathic POF (23). The following review article presents the role of autoimmunity in pathogenesis of POF, obtained by MEDLINE, EMBASE, Pub Med, Google Scholar, the Cochrane Library, and hand searches of pertinent references of English literature on POF and autoimmunity, cited between the January 2000 and December 2013. Reference lists of review articles, relevant trials, immunology and gynecology textbooks, and abstracts of scientific meetings were also searched. The literature search was performed using PubMed keywords: premature ovarian failure, premature ovarian insufficiency, premature ovarian dysfunction, hyper gonadotropic hypogonadisem, autoimmune oophoritis, and autoimmunity.

**Autoimmunity and POF**

The lack of a high sensitive and specific test has precluded accurate estimation of the prevalence of autoimmune type of POF (23, 29). Nevertheless, depended on some studies results, autoimmunity is responsible for approximately 4-30% of POF cases (5-33). The autoimmune involvement is base on the presence of antiovarian antibodies (AOAs), the histological evidences of lymphocytic oophoritis, and association with other autoimmune disorders (2, 18, 23, 34-42).

**AOAs**

The exact mechanism of autoimmunity in pathophysiology of this disorder remains obscure; probably the genetic or environmental factors initiate the activation of immune system (5, 23). A review of literature regarding POF with autoimmune involvement reveals that the presence of autoantibodies directed against ovarian tissues and elements, and their targets in cellular and molecular levels hold important issues (23, 35, 43-45). Nevertheless, it should be mention that alteration in cellular immunity such as macrophage and dendrite cells abnormalities, change in CD4+/CD8+ ratio, as well as inappropriate expression of class II MHC antigens by granulose cells has been seen in POF patients (28, 46-48).

Vallotton and Forbes were the first to detect AOAs in sera of POF patients (49). After that, numerous studies showed antibody-mediated mechanism for ovarian involvement (2, 18, 35, 36, 45). The presence of these antibodies is in association with increased risk of ovarian failure independent of reproductive hormonal levels (50). AOAs may be detected in serum prior to the onset of clinical presentation of POF (51).

The antibodies binding to the various steroid hormone-producing cells (52-56), gonadotropins and their receptors (57-60), zona pellucida (61, 62), oocyte (63, 64), corpus luteum (23, 65, 66), and several other antibodies such as anticardiolipin and antinuclear antibodies (34, 43) have been reported as the markers of ovarian autoimmunity. The multiplicity of the suspected auto antigens and related antibodies illustrates the variety of pathologic process causing ovarian damage.

The antibodies directed against steroid-producing cells of various endocrine glands such as adrenal cortex cells, Leydig cells of the testis, syncytiotrophoblast of the placenta, and theca cells of the ovary named as steroid cell antibodies (StCAs). These antibodies are polyclonal immunoglobulins of the IgG class (67). Some enzymes involved in steroidogenesis pathways are the targets of these autoantibodies. The main antigenic targets of StCAs are P450-17α-hydroxylase (17α-OH), P450-side-chain cleavage (P450scc), and 21- hydroxylase (21-OH) (68). StCAs are present in some autoimmune diseases such as autoimmune polyendocrine syndromes (APS); type I, type II, Addison’s disease (AD), and POF (35, 69). Their prevalence is 60% in APS-I patients, 25-40% in APS-II patients, 60-87% in POF associated AD, and 3-10% in isolated POF (35, 43, 54-56). Reato *et al.* suggested that primary amenorrhea in association with StCAs leads to autoimmune background of the ovarian failure (55). Some studies demonstrated that the presence of these autoantibodies is a predictive marker for developing POF in patients with autoimmune AD (23, 55). It is likely that 17α-OH and P450scc are the main molecular targets of StCAs in sera positive patients with POF associated AD (23, 35, 54, 55, 68). Nevertheless, in sera of approximately 10% of these patients, neither P450scc nor 17α-OH antibodies were detected (35). This observation illustrates the presence of some unidentified autoimmune targets for StCAs. Falroni and colleagues found that the women with AD related POF are often (>91%) positive for one of three major immune markers of steroid–cell autoimmunity [17α-OH antibodies, P450scc antibodies, and 3β–Hydroxysteroid dehydrogenase (3β-HSD) antibodies]. In their study, only 3% of the patients with isolated POF are positive for these markers (35). These data are consistent with other studies that StCAS are not major antibodies in isolated POF or POF associated with non-adrenal autoimmune disease. These autoantibodies seem to be main serologic markers for ovarian failure in AD related POF patients (23, 54, 70-72). Falroni and colleagues also showed that 3β-HSD is not a major auto antigen in autoimmune POF. They concluded that autoantibodies against this enzyme have limited application in routine clinical practice. In fact, the presence of 3β-HSD autoantibodies could be the secondary consequence of activation of immune system (35). In contrast with this study, Arif *et al. *suggested that 3β-HSD autoantibodies have a higher diagnostic sensitivity than other StCAs for autoimmune POF (73). The discrepancy between these studies could be explained in part by the differences in laboratory techniques used to measure antibodies; radio binding assay with in vitro translated recombinant 35S-labelled auto antigens for Falroni and immunoblotting technique for Arif (35, 73).

In earlier published studies, some autoantibodies have been detected on the surface of granulose cells by indirect immunofluoresence. The researchers have suggested that gonadotropin receptors are the targets for these antibodies (30, 63, 74). The inhibition of biological activity of gonadotropins and the decline in binding capacity of gonadotropins to their receptors are two main suggested mechanisms (75, 76). The findings of the earliest studies regarding the role of these autoantibodies in development of autoimmune POF were not supported by subsequent researches (58, 77). In other way, anti-gonadotropin receptor antibodies were also detected in iatrogenic ovarian failure (37). In fact, the real significance of these antibodies remains to be determined (78).

In order to detect autoantibodies directed against gonadotropins, some researchers investigated serum factors that might be able to inhibit FSH or Luteinizing hormone (LH) activity. Gobert and colleagues demonstrated the existence of anti-βFSH antibodies in sera of POF patients (57), their clinical significance, and diagnostic relevance still needs to be investigated through well-designed diagnostic studies.

Zona pellucida (ZP) is an extracellular matrix surrounding the mammalian oocyte. Its molecular structure consists of glycoproteins with strong antigenic potency. Takamizawa *et al.* detected anti-ZP antibodies in sera from idiopathic POF patients. They introduced a new microdot assay with high specificity for detecting anti-ZP antibodies (79). Their results are in agreement with the various independent reports on the existence of anti-ZP antibodies (61, 62, 80). The suspected pathological effect is the impaired communication between oocyte and granulose cells (62). At present time, there are no well-designed diagnostic studies to demonstrate the prevalence of these antibodies in POF patients. Thus, the exact importance of these autoantibodies is still unclear.

Anti-oocyte cytoplasm antibodies have been detected in patients with POF (63, 64). Pires and his group demonstrated that the cytoplasm of oocyte probably contains the most autoimmune targets in POF patients (78, 81). Although, the exact nature of the antigenic targets are still unclear, MATER (Maternal Antigen That Embryo Require), a 125KDa protein may be a possible candidate (26, 82-84). Very little is known about the precise nature of this protein. We need further studies to provide information about MATER and aid in deciphering its role in ovarian biology. The other identified antigens are Aldehyde dehydrogenase1A1 (ALDH1AI), Selenium Binding Protein 1 (SBP1), α-enolase, and Heat Shock Protein 90 (HSP90) (70, 81, 85, 86). According to Pires *et al*. study, HSP90ß is the most immunodominant antigen (78, 81). In contrast with these studies, the autoantibodies against oocyte were also found in sera of healthy individuals and a number of neoplastic, autoimmune, and inflammatory diseases (86-91).

In various studies, AOAs were detected by different methods (eg; enzyme-linked immunosorbent assay, immunohistochemistry, Western blotting, and indirect immunofluorescence). The most common used methods are indirect immunofluorescence (IIF) and enzyme-linked immunosorbent assay (ELISA) (23). Few studies have considered the measuring of autoantibodies against 21-OH by immunoprecipitation assay equivalents to measurement of these autoantibodies by (IIF) (92, 93). In the other way, Novosad *et al*. showed that validity of immunofluorescence for detecting AOAs is questionable (29).

The prevalence of AOAs in POF women varies greatly, ranging from 3 to 66.6 percent (23, 28, 35, 94, 95). These conflicting results could be explained by the difference in design elements of studies such as criteria for study and comparison groups, sample size, method of antigen preparation and antibody detection (2, 10, 18, 23, 94, 96). We suppose that the assessment of only one of several antigenic targets in some of studies, the differences in antigen sources, heterogeneity of histological targets, selection of animal or human ovaries at different ages or at different periods of menstrual cycle, and transient appearance of some of antiovarian antibodies have also changed the outcome of above-mentioned experiences.

AOAs might be the main cause of pathogenesis of autoimmune POF, or might be the result of ability of antibodies production by this autoimmune disease. In the other hand, there is poor correlation between antibody levels and severity of disease (18). Some authors conclude that clinical diagnosis of autoimmune POF, exclusively based on the detection of presence or absence of AOAs will be a great mistake (2).

A major weakness in the assessment of the role of AOAs is the high rate of false positive results (poor specificity) (29). These autoantibodies could be found in significant numbers of control groups (13, 29). It might be due to the presence of naturally occurring antibodies (NAA) (97). 

At present time, there is no valid serum marker to certainly prove the diagnosis of autoimmune POF.

**Lymphocytic oophoritis and POF**

The histopathological evidences of autoimmune ovarian involvement have been illustrated in 9.1-11% of the samples of ovarian biopsies belong to normal karyotype women with hyper gonadotropic amenorrhea (37, 40). Autoimmune oophoritis is usually detected in AD associated POF patients. Rarely, this pathologic picture was seen in association with isolated POF (14). Cellular infiltration of follicles by macrophages, natural killer cells, T-lymphocytes, plasma cell, and B-lymphocytes is the characteristic sign of an autoimmune oophoritis. The steroid-producing cells are the main target of autoimmune attack (18, 98). In some cases, abnormal activation of epithelial cells and subsequent follicular depletion and fibrosis were seen (98). Nevertheless, the specific histological picture is sparing of primordial and primary follicles and involvement of developing follicles by lymphocytic infiltration of theca cells (56, 99, 100). With respect to the multifollicular appearance of ovaries in this condition, the size of the involved ovaries could be normal or enlarged on sonographic view (56). Although, follicular depletion is the final stage of this autoimmune attack, the histological samples of ovarian tissues reveal the developing follicles in varying sizes in the majority of patients (101, 102). This observation supports the positive effect of immunosuppressive agents in restoring ovarian functions.

Massin and colleagues showed pelvic ultrasonography and hormonal profile (FSH, LH, estradiol, inhibin B serum levels) are insufficient to predict the presence or absence of follicular structures in POF patients with normal karyotype. They recommended ovarian biopsy as the reliable assessment for detecting follicle presence and activity in these patients (102). Ovarian biopsy is the gold standard for detecting autoimmune involvement of ovarian tissue and the presence of developing follicles in involved ovary (28, 37, 40). Nevertheless, this procedure is invasive and expensive. In other way, few studies demonstrated that the ability of ovarian biopsy in representing of follicular density of the whole ovary is questionable (10, 14, 103). We need a noninvasive screening test to avoid unnecessary ovarian biopsy and to select the women with possibility of autoimmune oophoritis. The antibodies to steroid–producing cells have been frequently found in the patients with histological evidences of lymphositic infiltration of the ovarian tissue (37, 54). Bakalov *et al*. showed a positive correlation between the presence of autoimmune oophoritis and serum adrenal cortex antibodies. In light of this observation, they recommended the measuring of adrenal cortex autoantibodies in sera of POF patients to select the patients with possibility of autoimmune oophoritis (56). Unfortunately, we did not find the published placebo-controlled randomized clinical trials with appropriate powers to support this finding.

**POF in association with other autoimmune diseases**

Ovary is a common target of autoimmune attack in organ-specific and systemic autoimmune diseases (18, 23, 54, 89, 104, 105). Approximately 10%-55% of patients with POF have associated autoimmune disorders (18, 23). Hypothyroidism is the most common associated autoimmune disorder with POF (25-60%) (3, 106, 107). Coincidence with diabetes mellitus is 2.5% (18). Autoimmune adrenal insufficiency may present as an isolated disease or may be associated with other autoimmune disorders (68). Approximately 10-20% of Addison patients have POF, and 2.5-20% of women with POF showing some evidences of autoimmunity against the adrenal gland (18, 55, 108). Dal Pra *et al*. showed no difference in the age at onset of POF in patients with or without Addison’s disease (54). Autoimmune POF usually occurs before the onset of adrenal involvement (23). In Reato *et al*. study, AD preceded POF. Nevertheless, the mean ages at onset of each disease were close (27 years for AD and 28.5 years for POF) (55). The association of POF with AD could be due to the presence of cross-reacting autoantibodies that react against auto antigens common to steroid producing cells with different origins (54, 55, 108). StCAs were detected in 60-87% of POF-associated adrenal involvement and 3-10% of patients with isolated form of this disorder (35, 37, 43, 52-56). The detection of antibodies against 17α-OH and P450scc in Addison patients is a predictor factor of for development of POF (68, 92). In the other hand, some authors suggested that the presence of 21-OH antibodies in sera from women with autoimmune POF might be important marker in identifying patients at risk of developing autoimmune adrenal insufficiency (35, 54, 68, 92). As already recognized by above mentioned reports, the POF patients with positive sera for StCAs are at risk for adrenal insufficiency, a potentially fatal condition, especially during pregnancy (10, 13, 35, 68, 108). Untreated adrenal insufficiency could be associated with serious fetal and maternal complications such as postpartum adrenal crisis (13, 109). Therefore, identification of this subgroup of POF patients with subclinical adrenal insufficiency is essential before decision making for egg or embryo donation. Bakalov *et al*. suggested that the morning serum cortisol level has low sensitivity and specificity, when used as a screening test in POF patients. Based on their findings, the measuring of adrenal cortex autoantibodies by indirect immunofluorescence technique or antibodies against 21-OH by immunoprecipitation assay are effective screening tests to detect asymptomatic adrenal insufficiency in these patients (108). It is in agreement with the previous reports on this subject (71, 92, 108). They also recommended that the presence of adrenal insufficiency could be confirmed by the standard ACTH stimulation test (108). We suggest referral of POF patients with positive adrenal cortex antibodies to endocrinologist for additional evaluations and long–term follow-up of adrenal functions. We also propose routine screening of thyroid function and glucose tolerance in POF patients. Due to the low frequency of the other associated autoimmune diseases, wider endocrine function screening is not recommended.

Autoimmune polyglandular syndromes (APS) are a series of disorders characterized by autoimmunity against two or more endocrine organs (10, 27). APS-I is a rare autosomal recessive disorder caused by mutation in the *AIRE* (autoimmune regular) gene. The role of this gene is regulation of immune tolerance (105). POF develops in 41-72% of patients with APS type I (13, 18, 55, 101, 104). Gonadal failure tends to appear at a younger age and in the highest prevalence compared with the other forms of APS (54, 55, 68). This event could be due to the mutations of *AIRE* gene in patients with APS-I (54, 55, 68). APS-II is an autosomal dominant disease. The prevalence of ovarian failure in APS-ll is 10-25% (55, 105, 110). Autoantibodies directed against steroidogenic enzymes and ovarian steroid–producing cells mediate ovarian dysfunction (111). In general, AD precedes POF in patients with APS-I, and follows POF in those with APS-II (55). Reato and colleagues suggested that StCAs, 17α-OHAb, and P450sccAb are the immunological predictive markers of potential autoimmune POF in the patients with autoimmune Addison’s disease and the different types of APS (55).

A review of literature regarding the relation between POF and autoimmunity indicates that autoimmune involvement is the main mechanism of POF in cases associated with other autoimmune diseases, but in isolated POF the evidences supporting this subject is weak (23, 43). However, the higher anti-Mullerian hormone and inhibin levels, a low incidence of histological evidences of oophoritis, and frequent reports of spontaneous pregnancy in isolated autoimmune POF could be the best evidences of transient ovarian damage and some degrees of function preservation (24, 112, 113). We need the placebo-controlled randomized clinical trials to determine the true roles of autoimmunity in pathogenesis of isolated POF.

**Infertility and autoimmune POF**

The involvement of autoimmune mechanisms have been suggested in various ovarian pathology, such as polycystic ovarian syndrome, unexplained infertility, recurrent pregnancy loss, endometriosis, and other conditions with poor reproductive outcome (14, 23, 30, 43, 51, 114).

Numerous studies illustrated that standard treatment outcome for infertility is less effective in the presence of ovarian autoimmunity. The presence of anti-oocyte cytoplasm antibodies in follicular fluid was associated with unsuccessful oocyte retrieval and fertilization failure in IVF patients (115). Some studies demonstrated association between the presence of serum AOAs and lower pregnancy rate in women seeking infertility treatment (34, 51, 116). Furthermore, a high correlation was seen between poor response to IVF programs and the presence of AOAs in sera of infertile women (34, 117, 118). Monnier-Barbarino and colleagues suggested that hormonal stimulation and ovarian trauma during follicular puncture could be the causes of developing or enhancing of AOAs formation in patients with history of repeated IVF attempts (117). In another study, the immunization process against exogenous gonadotropins and the development of anti-FSH and anti-LH antibodies were considered as the cause of poor responding to IVF program (118). In the other hand, some authors suggested that anti-ZP antibodies are one of the causes of IVF failure by their blocking effects on sperm-ZP binding and sperm penetration (79, 119-122). In light of this observation, Mardesic *et al*. recommended intracytoplasmic sperm injection (ICSI) as the method of choice in anti-ZP antibody-positive infertile couples (123). Pires proposed the evaluation of AOAs as a prognostic factor in patients undergoing IVF-ET program (78). Later on, Khole recommended that serological assessment of AOAs before starting IVF protocols would be justified in poor responder patients (2).

With respect to the possibility of the presence of AOAs in sera of POF women years before the onset of clinical presentation as well as detection of these autoantibodies in one third of patients with unexplained infertility (95), some authors suggested this form of infertility as an early stage of autoimmune POF. They also recommended the evaluation of ovarian autoimmunity as part of an infertility workup to predict the chance of development of gonadal failure in future and identify the best candidates for oocyte retrieval before follicular depletion (23, 70). In fact, we have little information about the predictive value of AOAs in ovarian reserve assessment. We need well-designed studies to clarify this subject.

**The effect of Immunosuppressive treatments**

Lymphocytic infiltration of theca layer of developing follicles and sparing of primary and primordial follicles presents the possibility of resumption of ovarian function by immunosuppressive treatments (99, 100).

Although in most instances, treatment with immunosuppressive agents failed to reverse the course of the ovarian autoimmunity or enhance the ovarian response to gonadotropins (18, 124). Numerous studies have recommended cell-mediated and humoral immunity suppression by glucocorticoids or anti-B-cell therapies for reversal of infertility or resumption of ovarian function in the selected groups of patients with autoimmune POF (101, 111, 125-128). It should be in mind that none of these studies was randomized controlled trial with well-selected autoimmune POF patients (129). Indeed, these reports were mostly case series studies (127, 130, 131). On the other hand, some patients developed serious complications such as Cushing syndrome and knee osteonecrosis after receiving corticosteroids (132). Bats *et al*. recommended tissue diagnosis of local autoimmune reaction by ovarian biopsy before starting immunosuppressive agents (98). Although, the gold standard for detecting autoimmune POF is ovarian biopsy (28, 37, 40), this procedure is not recommended due to unknown clinical value, expense, and risks (10, 14, 103). With respect to the association between the presence of adrenal cortex antibodies and autoimmune oophoritis, the measuring of these autoantibodies in serum has been proposed as noninvasive screening test to avoid unnecessary ovarian biopsy and select the patients with possibility of autoimmune oophoritis (56).

Dehydroepiandrosterone (DHEA) is an endogenous steroid agent. Zona reticularis layer of the adrenal cortex and ovarian theca cells are sources of secretion in healthy women. This molecule is precursor of testosterone, androstenedione, and estradiol (133). DHEA promotes activation of the oocytes and inhibits atretic phenomena (134). Barad *et al*. noted higher pregnancy rates with DHEA supplementation in patients with diminished ovarian function (134, 135). Mamas and Mamas reported successful pregnancy following DHEA therapy in women with POF (130). Barad and colleagues believed that the patients in this study did not fulfill the criteria of POF. They recommend the termination of “premature ovarian aging” (POA) for definition of these patients with increased FSH levels and diminished ovarian reserve (135). We did not find a specific treatment modality for autoimmune oophoritis with proven efficacy and safety confirmed by prospective randomized placebo-controlled studies.

Other suggested fertility options in POF patients are IVM (in vitro maturation) of oocyte derived from primordial follicles or stem cells and IVF (in vitro fertilization) using donor gametes or embryos (10, 136).

## Conclusion

In conclusion, an abundance of evidence shows that autoimmunity is responsible for development of POF especially in cases associated with other autoimmune diseases. Several potential immune targets in various parts of ovary have been proposed for antibody-mediated autoimmune attack. The oocyte seems to be the most often targeted cell. Despite the considerable scientific work, the precise nature of molecular targets responsible for this autoimmune process remained unclear. The studies regarding antiovarian antibodies have conflicting results, partly due to differences in the detection methods, heterogeneity of the patients and control groups, and the lack of high specificity and sensitivity tests. This fact illustrates the need for accurate diagnostic tools in order to identification the specific antigenic targets and related autoantibodies.

Numerous studies illustrated that standard treatment outcome for infertility is less effective in the presence of ovarian autoimmunity. Subsequently, some authors suggested the possible effects of immuno-modulating therapy on the resumption of ovarian function and fertility in a selected group of POF patients. Nevertheless, in most instances, this treatment fails to reverse the course of the disease.

The further information regarding autoimmune actions of AOAs will lead to elucidate the underlying mechanisms of ovarian damage. It also will help the development of better approaches to diagnose, and effective therapeutic regiments. We also suppose that the specific noninvasive tests are essential to detect associated disorders, as well as to select the women with acceptable chance of restoring of ovarian function and fertility by immunosuppressive therapy.
